# Extensin network formation in *Vitis vinifera *callus cells is an essential and causal event in rapid and H_2_O_2_-induced reduction in primary cell wall hydration

**DOI:** 10.1186/1471-2229-11-106

**Published:** 2011-06-14

**Authors:** Cristina Silva Pereira, José ML Ribeiro, Ada D Vatulescu, Kim Findlay, Alistair J MacDougall, Phil AP Jackson

**Affiliations:** 1 Plant Cell Wall Laboratory, Instituto de Tecnologia Química e Biológica/Universidade Nova de Lisboa, Apartado 127, 2781-901 Oeiras, Portugal; 2 Cell and Developmental Biology Department, John Innes Centre, Norwich Research Park, Norwich NR4 7UH, UK; 3 Department of Food Biophysics, Institute of Food Research, Norwich Research Park, Colney, Norwich, NR4 7UA, UK

## Abstract

**Background:**

Extensin deposition is considered important for the correct assembly and biophysical properties of primary cell walls, with consequences to plant resistance to pathogens, tissue morphology, cell adhesion and extension growth. However, evidence for a direct and causal role for the extensin network formation in changes to cell wall properties has been lacking.

**Results:**

Hydrogen peroxide treatment of grapevine (*Vitis vinifera *cv. Touriga) callus cell walls was seen to induce a marked reduction in their hydration and thickness. An analysis of matrix proteins demonstrated this occurs with the insolubilisation of an abundant protein, GvP1, which displays a primary structure and post-translational modifications typical of dicotyledon extensins. The hydration of callus cell walls free from saline-soluble proteins did not change in response to H_2_O_2_, but fully regained this capacity after addition of extensin-rich saline extracts. To assay the specific contribution of GvP1 cross-linking and other wall matrix proteins to the reduction in hydration, GvP1 levels in cell walls were manipulated *in vitro *by binding selected fractions of extracellular proteins and their effect on wall hydration during H_2_O_2 _incubation assayed.

**Conclusions:**

This approach allowed us to conclude that a peroxidase-mediated formation of a covalently linked network of GvP1 is essential and causal in the reduction of grapevine callus wall hydration in response to H_2_O_2_. Importantly, this approach also indicated that extensin network effects on hydration was only partially irreversible and remained sensitive to changes in matrix charge. We discuss this mechanism and the importance of these changes to primary wall properties in the light of extensin distribution in dicotyledons.

## Background

The central role that the primary cell wall plays in regulating extension growth, cell adhesion and cell morphology, requires a tight temporal-spatial regulation of its rheological properties, which are ultimately determined by matrix composition and structure. Most current primary cell wall models agree that the major wall polymers are bound to each other largely non-covalently, although physically intertwined [1,2]. In these models, hemicellulose is associated with cellulose through hydrogen bonding and physical entrapment, and pectins form a relatively mobile gel around the cellulose-hemicellulose network or between cellulose-hemicellulose lamellae [3,4]. In some tissues of dicotyledons, extensins are abundant and are also thought to play an important role in primary wall biosynthesis [5-7] and to contribute to their structural properties [8]. Although the composition and structure of the major matrix polymers in dicotyledons have been well characterised, understanding how changes in polymer compositions and their interactions in the matrix nanostructure relate with changes in wall properties remains a challenge.

Plant cell expansion is ultimately driven by turgor pressure, but controlled by the cell wall ability to yield to tension stress [9]. Wall stress-relaxation during the integration of newly synthesised material into the matrix requires the co-ordinate action of matrix modifying enzymes including expansin [10], xyloglucan endotransglycosylase/hydrolase (XHT) [11], a variety of glycosyl hydrolases and possibly some class III peroxidases through hydroxyl radical production and the resultant scission of wall polysaccharides [12].

To oppose relaxation, the regulation of extension growth is thought to involve processes leading to a loss of wall plasticity, rather than a loss of turgor pressure [13]. Such processes include processive pectin methyl esterases which demethylate homogalacturonans (HGs) to promote Ca^2+ ^bridging and rigidification [14]. A borate diester cross-link between rhamnogalacturonan-II chains, which contributes to the tensile strength has been described (reviewed in [15]). In dicotyledons, there is evidence for the covalent cross-linking of pectin to xyloglucan [16] and pectin to the extensin network [17], which might also contribute. Class III peroxidases are also regarded as potentially important cell wall stiffening enzymes [18], since peroxidase/H_2_O_2_-driven reactions may fix the viscoelastically extended cell wall through phenolic cross-linking [19], which can occur between feruloylated pectins [20] or extensins [21,22].

Cell adhesion has been less studied, but there is evidence that this occurs primarily at the edges of cell faces bordering intercellular corners, rather than across the entire wall face [23]. The corners of intercellular faces thus formed can contain weakly esterified HGs [24], which can be cross-linked by Ca^2+^, leading to greater adhesive strength [14]. Support for this comes from recent descriptions of the Arabidopsis *tsd2*/*qua2 *mutant, which is defective in a putative Golgi-based (pectin) methyl transferase gene and shows a reduction in both HG content [25] and cell adhesion [26]. Extensin is also present in the intercellular spaces at cell corners in some tissues [6,27]. These structural proteins electrostatically interact with HGs, promoting pectin gelation [28], and are thought to promote further matrix rigidification after extensin network formation [7,29], with possible consequences to the strength of intercellular adhesion.

A further important, but often overlooked constituent of the cell wall is water, which can constitute ca. 75% of its weight and confers the properties of a relatively dense gel to the matrix [9]. Cell wall water content has been shown to have a direct effect on hypocotyl extensibility in sunflower [30]. Studies with dicotyledons have demonstrated that changes in cell wall hydration primarily affects the mobility of pectins and a minor fraction of the xyloglucan network, while cellulose and more tightly bound forms of xyloglucan remain as typical, rigid solids [31,32]. Although it is not yet clear if the relatively mobile pectin network can resist stresses in the plane of the wall, a decrease in the mobility of methyl-esterified pectin has been correlated with growth cessation in celery collenchyma [33]. It has also been suggested that pectins and xyloglucans could regulate the matrix free volume and viscosity to control microfibril realignment and extension growth [4,34]. Alterations to pectin mobility, through either changes in hydration or the formation of cross-links, could therefore be important to matrix and cell adhesive properties during development.

Primary cell walls are negatively charged at physiological pH due to the high abundance of charged HGs. The polyelectrolyte nature of HG-rich areas of the matrix can drive wall swelling through a Donnan effect, where increased hydration would occur as the concentration of endogenous counterions, such as Ca^++^, Mg^++ ^and K^+ ^are reduced in the apoplastic space [35]. Demethylation of HGs by pectin methyl esterases [36] can increase charge density in the matrix and therefore drive increased hydration. Conversely, the formation of calcium-pectate bridges may constrain matrix swelling [37]. In addition, the electrostatic interaction of basic peptides with pectins can increase pectin gelation by reducing pectin charge and hydration [28], indicating that the electrostatic interaction of wall proteins with the matrix is important.

Extensins can be abundant in dicotyledon primary cell walls (up to 10% w/w). These structural proteins have a poly(II) Pro-like configuration giving them a rod-like shape in solution [8], which can reach 50-100 nm in length [8,38]. The presence of highly periodic Lys-containing motifs in the primary structures of typical extensins promotes their electrostatic interaction with HGs [8,28], with possible consequences to pectin mobility and wall matrix swelling.

Monomeric extensin can also be covalently cross-linked within the extracellular matrix to an insoluble extensin network by a H_2_O_2_/peroxidase-mediated process [22,39,40], thought to be mediated by particular class III peroxidases referred to as extensin-peroxidases (EPs) [41,42].

Electron microscopy studies of the primary cell wall in onion have indicated thin walls, ca. 100 nm thick, composed of 3-4 laminae of 8-15 nm thick microfibrils coated with xyloglucan, and spaced 20-40 nm apart [1]. Consistently, recent AFM studies of potato cell walls have indicated an interfiber spacing of 26 nm [43]. These dimensions suggest that monomeric extensin can span inter-microfibrillar distances, and it is therefore conceivable that the formation of network extensin could help lock the major wall polymers to increase cell wall rigidity [8]. In fact, several studies suggest that extensin network formation is important for a wide range of plant physiological processes, including correct primary cell wall biosynthesis [5,7], cell adhesion and morphology [6], growth cessation [44] and disease resistance [45]. However, experimental data describing the effects of extensin network on primary wall properties has been lacking.

We have selected a grapevine callus line containing high amounts of monomeric extensin (GvP1) in the cell wall, which is insolubilised after the addition of H_2_O_2 _in a reaction exclusively catalysed by the EP, GvEP1 [22]. These cells provide a convenient system to evaluate the contribution of specific cell wall proteins, such as extensin and EP, to rapid, H_2_O_2_-mediated changes in cell wall properties. Using this system, we have demonstrated that extensin network formation drives a rapid increase in resistance to fungal lytic enzymes [29]. Here, we report that H_2_O_2 _can rapidly reduce the hydration and thickness of primary dicotyledon cell walls, and that extensin network formation is the primary and causal event in this process.

## Results

### Rapid, H_2_O_2_-mediated effects on cell wall hydration and thickness

**Figure 1:**
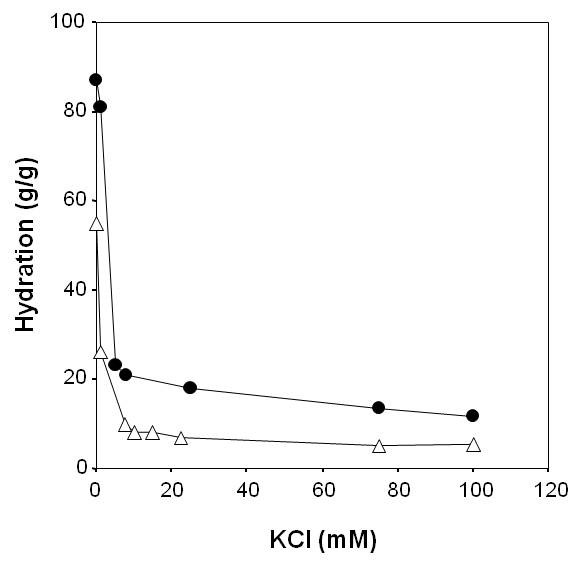
**Swelling behaviour of grapevine native cell walls at pH 4.5 as a function of KCl concentration**. Closed circle, control; open triangles, after incubation with H_2_O_2_.

The swelling behaviour of isolated cell walls from grapevine callus in solution with 0-100 mM KCl at pH 4.5 is typical of a weak polyelectrolyte (Figure 1, closed circles). A Donnan-type effect is observed in that cell wall swelling increases as the concentration of the counterion is reduced; an effect which is more pronounced for values below 10 mM KCl.

Following incubation with 100 μM H_2_O_2 _at pH 4.5 for 30 min, these walls retained the capacity to show increased swelling at reduced KCl concentrations, but demonstrated a remarkable reduction in hydration at all KCl concentrations (Figure 1, open triangles), indicating a rapid and H_2_O_2_-mediated formation of a denser matrix.

**Figure 2:**
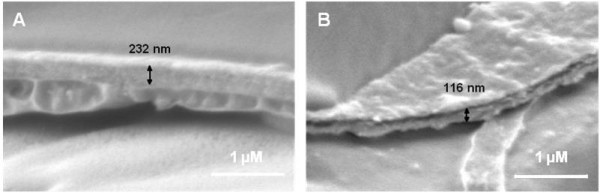
**Scanning electron micrographs of typical cell walls in fractured grapevine callus cells**. A) Untreated; B) incubated with H_2_O_2_. Scale bars equivalent to 1 μm are indicated in the bottom, right hand corners of the panels. Both samples were equilibrated in 15 mM KCl prior to freeze-fracture.

In order to determine if the changes in hydration occurred with alteration in cell wall dimensions, their thicknesses were measured by fast-freeze scanning electron microscopy (Figure 2). The measurements suggested the apparent cell wall thickness varied substantially between samples, possibly due to the occasional difficulty of identifying wall limits and of obtaining views precisely perpendicular to the cell wall plane. Nevertheless, measurements indicated that native cell walls at 0 mM KCl were on average ca. 230 nm thick (S.E. of ±. 20.1). The presence of 15 or 100 mM KCl resulted in a significant reduction to 180 and 174 nm, respectively (Student t-test p < 0.05, n ≥ 15). The incubation of cells pre-equilibrated in 15 mM KCl with H_2_O_2 _resulted in the formation of cell walls on average ca. 25% thinner, at 134 nm (Student t-test p < 0.01, n ≥ 15).

### H_2_O_2_-induced reduction in cell wall hydration is accompanied by GvP1 network formation

**Figure 3:**
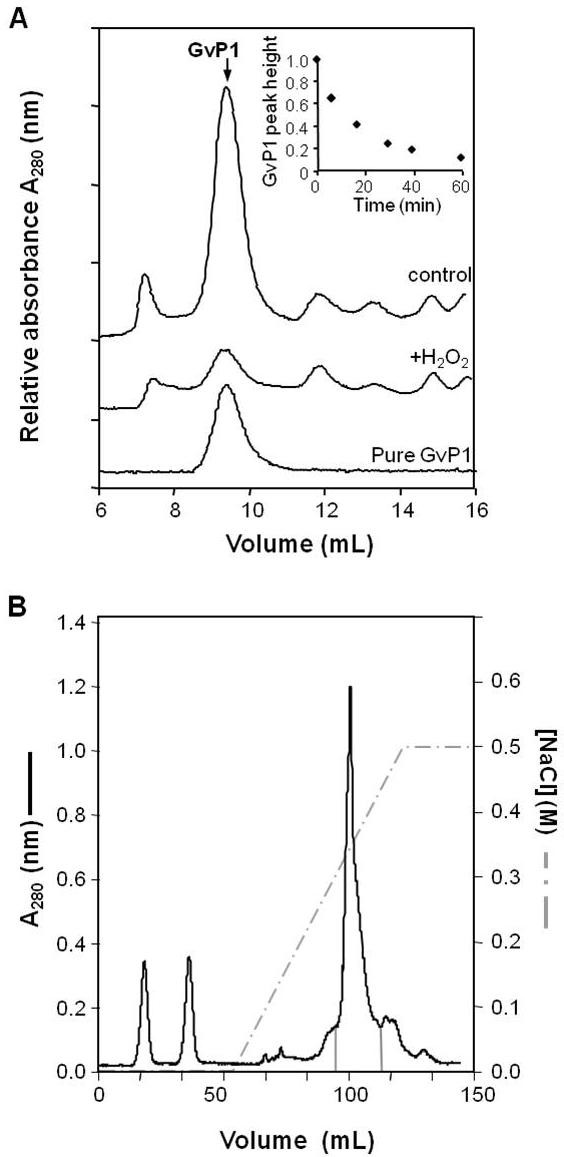
**H_2_O_2 _causes insolubilisation of the grapevine extensin, GvP1**. A) Superose-12 chromatography of EIBMPs (saline eluates of 35 mg (FW) cells) from untreated cells (trace control), cells incubated with 100 mM H_2_O_2 _(trace + H_2_O_2_). A chromatogram of pure GvP1 is also indicated as a reference. A time course assay of GvP1 insolubilisation *in muro *is depicted in the inset. B) SP-Sepharose chromatography of whole EIBMPs. Fractions enriched in GvP1 and subject to TCA fractionation for the purification of GvP1 are delimited by solid grey lines.

We have previously reported that grapevine callus cells contained high levels of a single monomeric extensin, GvP1 [22], which is uniformly distributed as a monomer in the lateral walls [29]. No other extensins were detected in extracts of these cells, and saline extraction of walls resulted in the near complete removal of JIM11 epitope signals, indicating minor, if any network extensin prior to incubation with H_2_O_2_. To determine if H_2_O_2_-mediated reduction in cell wall hydration occurred with extensin network formation, extracellular, ionically bound matrix proteins (EIBMPs) from native and H_2_O_2_-treated cell walls (Figure 3) were compared by Superose-12, gel-filtration chromatography (Figure 3A). The chromatograms demonstrate that incubation with 100 μM H_2_O_2 _at pH 4.5 leads to the insolubilisation of a major protein peak (GvP1) eluting at 9.5 mL. The time course of GvP1 insolubilisation was followed by monitoring changes in the peak height of GvP1 over time, and ca. 60% insolubilisation of GvP1 was seen to occur within 15 min (Figure 3A, inset).

SP-Sepharose chromatography (Figure 3B) of saline extracts and trichloroacetic acid precipitation of selected fractions (see also methods) enabled the recovery of purified GvP1 from the supernatant (Figure 3A). MALDI-TOF analysis of GvP1 indicated a molecular mass of 90 kDa, without any significant additional mass signals, indicating purity (data not shown). The amino acid composition of GvP1 is typical of dicotyledon extensins (Table 1). Furthermore, a comparison of the amino acid composition of saline-extracted cell walls before and after H_2_O_2 _treatment demonstrated that the insolubilisation of GvP1 occurs with an increase in the major amino acids of GvP1 extensin in the saline-insoluble, cell wall structure (Table 1), confirming its incorporation into the wall matrix as an insoluble network. Quantitatively, GvP1 extensin network was calculated to contribute ≥ 0.6% (w/w [DW]) in control cell walls, but ca. 6% (w/w [DW]) of the cell wall weight after incubation with 100 μM H_2_O_2 _over 30 min.

**Table 1:** H_2_O_2_-mediated changes in the amino acid composition of saline-insoluble protein in the cell wall matrix

**a.a**.	GvP1	Control walls	H_2_O_2_-treated walls	H_2_O_2_-induced changes in walls
	**(mol %)**	**(nmole.mg^-1^)**	**(nmole.mg^-1^)**	**(nmole.mg^-1^)**

Hyp	45.3	3.0	32.6	29.6
Lys	12.0	6.1	17.7	11.6
Tyr	8.4	9.5	14.9	5.3
Ser	8.0	37.5	43.5	6.0
Pro	5.7	30.8	36.0	5.2

The amino acid (a.a.) composition of pure grapevine extensin, GvP1 (mol %), is shown for comparison. The content of each amino acid in wall preparations was measured after saline extraction to remove saline-soluble EIBMPs and is given in nmole.mg^-1 ^(DW) cell wall. The less abundant amino-acids of GvP1 are omitted for clarity.

### GvP1 displays characteristics typical of extensins

**Figure 4:**
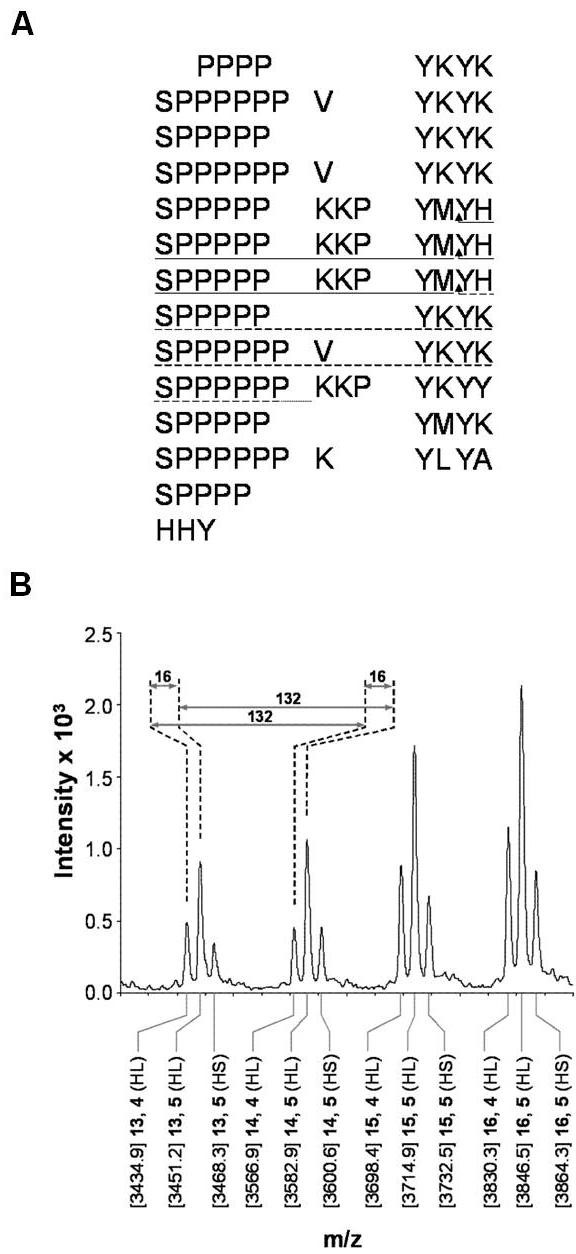
**GvP1 shows characteristics of typical, dicotyledon extensins**. A) Partial sequence of GvP1 deduced from its 5' truncated cDNA. Sequences obtained by Edman sequencing of isolated GvP1 peptides P4 (dashed line) and P6 (solid line) are indicated. CNBr cleavage sites are indicated by arrows. B) MALDI-TOF MS of Peptide 6 demonstrates mass heterogeneity due to variable arabinosylation (periodicity of 132 Da) and hydroxylation of proline residues (periodicity of 16 Da). The differing ion species are labelled along the x-axis with: [mass] number of Ara, number of Hyp (its identity as a homoserine (HS) or homolactone serine (HL) cleavage product).

To determine if GvP1 is a typical extensin, homology-based cloning (see methods) was utilised to isolate a 5' truncated extensin cDNA from grapevine callus. All ten clones sequenced encoded the extensin primary structure depicted in Figure 4A, or truncated versions of the same. This supports earlier results indicating the expression of a single extensin in these cells [22]. Cyanogen bromide cleavage of purified GvP1 enabled the isolation of two internal peptides (P4, P6) which were sequenced by Edman degradation. Both sequences could be localised within the extensin cDNA obtained (Figure 4A), confirming that it corresponded to GvP1. The sequence of GvP1 contains motifs typical of dicotyledon extensins, including repeats of structural Ser (Hyp)_4 _motifs, as well as Tyr-Lys-Tyr-Lys and Pro-Pro-Val-Tyr-Lys motifs believed to be required for the intra- and inter-crosslinking of extensin *in muro *[46]. However, an unusual sequence characteristic of GvP1 is the variable extension of the Ser (Hyp)_4 _motif to Ser (Hyp)_4-6_, resulting in a lack of the high frequency sequence periodicity present in many extensins [7]. Further evidence for GvP1 as a typical dicotyledon extensin comes from the MALDI-TOF/MS analysis of the 13 a.a. glycopeptide, P6 (Figure 4B). This peptide demonstrates a considerable mass heterogeneity, but with periodicities of 16 and 132 Da. This can be clearly attributed to the expected heterogeneity in proline hydroxylation (16 Da) and hydroxyproline arabinosylation (132 Da) in extensins.

### Saline-eluted walls regain their ability to reduce hydration in response to H_2_O_2 _when reconstituted with EIBMPs

**Figure 5:**
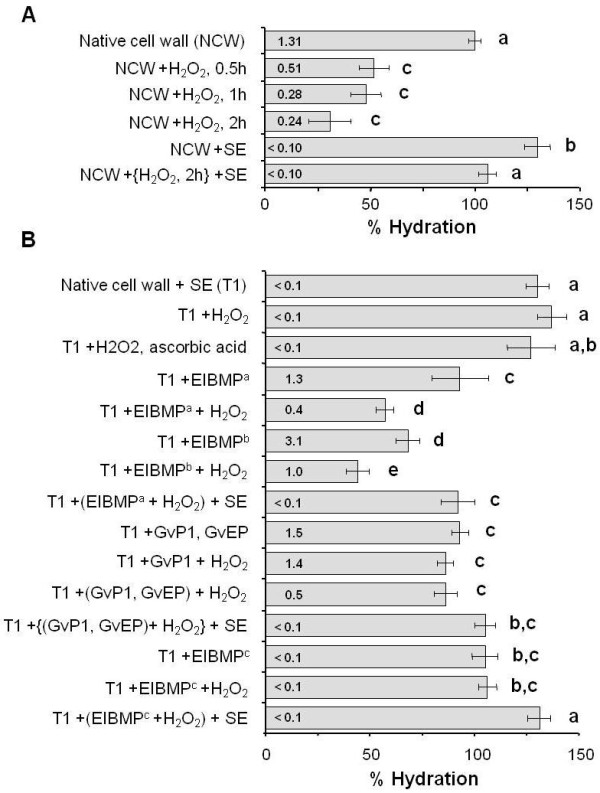
**The effects of H_2_O_2_, GvP1 extensin and EIBMPs on primary cell wall hydration**. A) The effect of H_2_O_2 _on native cell wall (NCW) hydration. B) Extracellular, ionically binding matrix proteins (EIBMPs) influence the effect of H_2_O_2 _on cell wall hydration. Saline extracted, native cell walls (treatment 1; T1) were used as the starting material for these experiments. Hydration measurements are presented as % hydration of native cell walls ± s.d. Each data point was calculated from the average of 4 samples, each measured in triplicate. Incubation with H_2_O_2 _was for 0.5 h, unless otherwise indicated. Amounts of saline-soluble GvP1 (μg.mg^-1 ^cell wall [FW]) after treatments is indicated within each bar. Student *t*-test was used to identify significant (*p *≤ 0.01) differences between hydration values. Key: Successive '+' symbols describe the order of treatments except those enclosed by brackets which were made simultaneously; SE = saline extraction; EIBMP^a ^= endogenous levels of whole EIBMPs; EIBMP^b ^= 2 × endogenous levels of whole EIBMPs; EIBMP^c ^= GvP1-free EIBMPs fractionated from native cell walls.

GvP1 network formation and changes in cell wall hydration were studied over time. These and all subsequent measurements of hydration were made at 15 mM KCl, where H_2_O_2_-mediated differences in hydration were marked. In native cell walls, > 60% of monomeric GvP1 was insolubilised after 30 min incubation with H_2_O_2 _with a ca. 50% reduction in cell wall hydration. Longer times of incubation resulted in higher levels of network formation and lower levels of cell wall hydration (Figure 5A), suggesting a causal relationship.

Importantly, the removal of EIBMPs by saline extraction was seen to increase hydration in control (native) cell walls, suggesting that the electrostatic interaction of endogenous matrix proteins with the wall is also an important determinant of wall hydration. Following H_2_O_2_-mediated partial dehydration, saline extraction was also seen to increase wall hydration, although to a significantly less extent to that seen after the saline extraction of control cell walls (Figure 5A).

Saline-extracted native walls showed no significant change in hydration in response to H_2_O_2 _or H_2_O_2 _plus ascorbate (Figure 5B), indicating that the presence of EIBMPs *in muro *was essential for H_2_O_2_-mediated changes in hydration.

In order to examine the role of specific EIBMPs in H_2_O_2_-mediated cell wall dehydration, we manipulated endogenous levels of selected EIBMPs, including GvP1, *in muro*. Saline-extracted grapevine callus walls (1 mg DW) retained the capacity to bind endogenous levels of total EIBMPs (70 μg) and GvP1 (50 μg; Additional file 1A, B). The binding of non-extensin EIBMPs (20 μg) is shown in Additional file 1C-D. This suggests that we can bind endogenous levels of selected EIBMPs to saline-extracted cell walls and assay for changes in hydration in response to added H_2_O_2_.

The use of similarly high salt conditions partially dissociates the pea xyloglucan-pectin interaction [47], suggesting this treatment could irreversibly alter the structure and/or composition of grapevine callus cell walls, with possible consequences to wall hydration. Analyses of neutral monosaccharide and uronic acid contents of different cell wall isolates (Table 2) did in fact indicate that saline extraction led to some loss of pectin (seen as a decreased content of uronic acids and arabinose). However, despite this apparent loss, the increase in wall hydration observed after saline-extraction could be completely reversed by the replacement of EIBMPs to endogenous levels (Figure 5B). The incubation of these manipulated cell walls with H_2_O_2 _resulted in both extensin network formation (Additional file 1A) and a decrease in hydration (Figure 5B) to levels comparable to those observed after H_2_O_2_-treatment of native cell walls (Figure 5A).

**Table 2:** Carbohydrate composition (mol %) of native and H_2_O_2_-incubated cell walls, with and without salt extraction

Cell Wall Isolate	mol % composition							
	**Rha**	**Fuc**	**Ara**	**Xyl**	**Man**	**Gal**	**Glc**	**UA**

Native	0.9	0.4	11.8	2.8	1.4	4.2	57.7	20.8
+saline extracted	1.0	0.5	4.9	3.1	1.5	4.1	68.7	16.1
+H_2_O_2_	1.2	0.5	12.3	3.0	1.5	4.4	54.9	22.3
+H_2_O_2 _+saline extracted	1.0	0.5	6.3	3.0	1.4	4.0	67.8	16.0

Rha = rhamnose; Fuc = fucose; Xyl = xylose; Man = mannose; Gal = galactose, Glc = glucose and UA = uronic acid.

The effects of the interaction of EIBMPs with the wall matrix interaction on hydration appear to be concentration dependent, since the addition of higher levels (2×) of EIBMPs resulted in a greater reduction in hydration prior to, and following H_2_O_2 _treatment. As in native cell walls, H_2_O_2_-mediated dehydration could be only partially reversed by the extraction of EIBMPs from the matrix by saline elution (Figure 5B).

Endogenous EIBMPs therefore must play an important role in determining the level of hydration in primary cell walls, through both their electrostatic interaction with the matrix and their apparent role in the further reduction of wall hydration in response to H_2_O_2_. These data also confirm that we can extract EIBMPs with high salt solutions, and subsequently re-bind them to the wall matrix, without irreversibly altering the wall's capacity to reduce hydration in response to H_2_O_2_. This convenient experimental system was therefore used to investigate the role of specific EIBMPs in this process.

### Effects of extensin network formation on wall hydration is reduced in the absence of other EIBMPs

The addition of purified GvP1 alone, or together with the extensin peroxidase, GvEP1, to saline-extracted cell walls was effective in reducing wall hydration to levels found in native cells (Figure 5B). No deposition of GvP1 was detected in response to H_2_O_2 _in cell walls without the extensin peroxidase, GvEP1. Where GvEP1 was present, H_2_O_2 _treatment resulted in the deposition of ca. 65% of extensin (see also Additional file 1B). However, the extensin network formation in these walls was not accompanied by any significant reduction in hydration (Figure 5B). Similarly, the addition of GvP1-free EIBMPs to saline-extracted walls reduced hydration to control levels, but no change in hydration was seen after the addition of H_2_O_2_. This is in contrast to the substantial reduction in wall hydration (50%) obtained after H_2_O_2 _incubation of walls containing total, EIBMPs (Figure 5A, B) and strongly suggests that the presence of EIBMPs other than GvP1 and GvEP1 is a pre-requisite for H_2_O_2_-induced reduction in wall hydration.

Nevertheless, whereas saline-extraction of untreated native walls resulted in substantial swelling, saline extraction after extensin network formation resulted in significantly less swelling. This smaller increase in hydration after extensin network formation was seen after H_2_O_2 _treatment of native walls (Figure 5A), or in saline extracted walls where either total EIBMPs or extensin and GvEP1 had been replaced (Figure 5B). This effect was restricted to walls which contained network GvP1, since saline extraction of H_2_O_2_-treated walls containing GvP1-free EIBMPs swelled to hydration levels similar to that observed after saline extraction of native walls (Figure 5B). The formation of the extensin network can therefore be considered to be effective in restraining further cell wall swelling.

### The addition of EIBMPs to GvP1 network-containing walls mimics H_2_O_2 _effects on wall hydration

**Figure 6:**
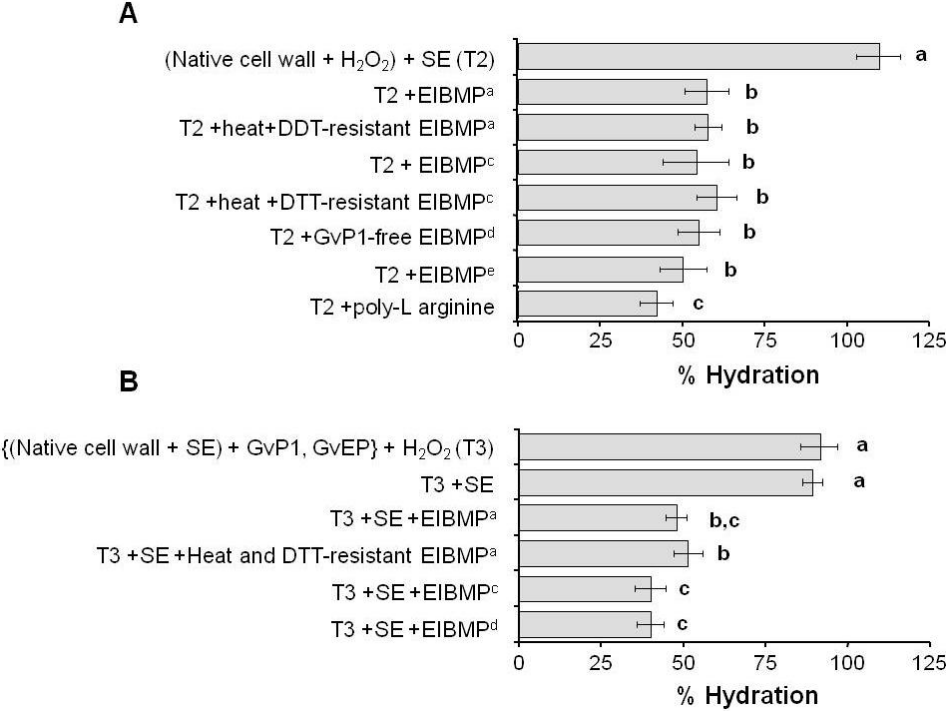
**The effect of selected fractions of EIBMPs on wall hydration in walls containing GvP1 network**. Where A) GvP1 network (ca. 70% deposition) was formed in the *presence *of total endogenous EIBMPs (T2) and B), GvP1 network (ca. 60% deposition) was formed with pure GvP1 and GvEP1, i.e. in the *absence *of other, endogenous EIBMPs (T3). In all cases, following extensin network formation, residual monomeric extensin was removed from walls by saline extraction prior to the addition of selected protein fractions. All measurements were made and expressed as described in Figure 5. Key: EIBMP^a ^= endogenous levels of whole EIBMPs; EIBMP^c ^= GvP1-free EIBMPs fractionated from native cell walls; EIBMP^d ^= GvP1-depleted EIBMPs from H_2_O_2_-incubated cell walls (see methods); EIBMP^e ^= Medicago leaf extracellular, ionically binding matrix proteins; DTT = dithiothreitol. Figure legend text.

To further investigate how the GvP1 network and other EIBMPs contribute to H_2_O_2_-mediated reduction in wall hydration, walls were prepared containing control levels of network GvP1, but free from non-extensin EIBMPs. In one approach, this was achieved by saline extraction of H_2_O_2_-treated native cell walls. The extensin network in such walls was, as a consequence, formed in the presence of endogenous EIBMPs (Figure 6A). The successful re-attachment of endogenous levels of EIBMPs (20 μg mg^-1 ^cell wall (DW)) obtained from H_2_O_2_-incubated native walls (contain GvP1-depleted EIBMPs) markedly reduced the cell wall hydration to ca. 55% (Figure 6A), i.e. to levels comparable to that observed after the incubation of native walls with H_2_O_2_. Quantitatively similar results (55-60%) were also obtained after the addition of endogenous levels of GvP1-free EIBMPs obtained after fractionation of saline eluates of native cell walls, clearly indicating that non-extensin EIBMPs do not require reaction with H_2_O_2 _to be effective. Similar data was obtained in a second approach, where the extensin network was formed in saline-extracted cell walls, i.e. in the absence of other EIBMPs (Figure 6B). These cell walls also contracted to ca. 50% volume after the addition of endogenous levels of whole, or GvP1-depleted EIBMPs.

Hydrogen peroxide-mediated reduction in primary wall hydration therefore appears to require extensin network formation, but is influenced by the electrostatic interaction of EIBMPs with the wall matrix.

In an attempt to define the nature of the non-extensin EIBMPs involved, heat and DTT-resistant proteins of saline extracts were isolated and assayed in extensin network-containing walls, and found able to reduce hydration to levels comparable to that achieved with total EIBMPs (Figure 6A, B). Saline-extracted cell walls were also able to bind 20 μg. mg^-1 ^cell wall (DW) of *Medicago *leaf cell wall proteins. As shown in Additional file 1D, the chromatographic profile of these saline-soluble proteins was not altered by incubation of the walls with H_2_O_2_, suggesting the absence of abundant cross-linking structural proteins. Poly-L-arginine (MW ca. 15 kDa) could also be bound to saline-extracted walls at 10 μg. mg^-1 ^cell wall (DW). For both *Medicago *cell wall proteins and poly-L-arginine, these added quantities reduced the wall hydration of saline-extracted walls to levels similar to that of native walls (100 ± 9%, 90 ± 12%, respectively. See also Additional file 2), and no significant changes in hydration were detected in response to H_2_O_2 _(data not shown). However, when the same amounts were added to extensin network containing walls, cell wall hydration was reduced to ca 50% and 40%, respectively (Figure 6A). No significant binding was obtained with poly-L-aspartic acid (MW ca. 11KDa), indicating the absence of cell wall sites for the ionic interaction with negatively charged polypeptides.

### Extensin effects on matrix hydration can be important in lateral walls and cell junctions

The effect of extensin network formation on primary wall hydration suggests that this post-translational process could impart important biophysical changes to extracellular matrix materials during development. Grapevine callus cell walls appear to have a monosaccharide composition typical of primary cell walls and GvP1 displays characteristics of dicotyledon extensins in general (Table 1 & Figure 4), suggesting the effect that network GvP1 has on primary wall hydration might also occur in other extensin-bearing primary cell walls during plant development.

**Figure 7:**
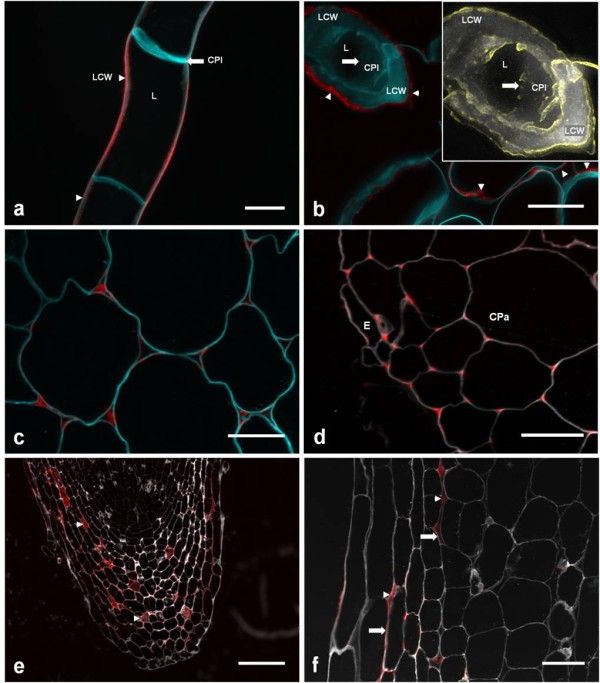
**JIM11 detection of extensin epitopes in selected tissues of grapevine, potato and Arabidopsis**. A) Confocal image of frozen callus. B) 0.5 μm sections of resin-fixed callus. *Inset*: Magnified image of calcofluor signal from transverse section of callus cell (from top left corner), in which edge detection (yellow) was used to highlight the spatial limit of the broken cell plate. N.B. JIM11 signals are in lateral cell walls (arrowheads), and not the cell plate (arrows). C) Cortical parenchyma of basal grapevine epicotyl. D) Epidermal and cortical parenchyma of root. E) Root cap. Note the presence of JIM11 epitopes in large intercellular spaces (arrow heads). F) Higher amplification of lateral outer layer of root cap. N.B. JIM11 epitopes are present in both intercellular spaces (arrow heads) and cell wall (arrows). Scalebars: A-F, 25 μm; G, 250 μm; H, 100 μm. In all cases, calcofluor (for cellulose marking) signals were false-coloured to cyan (panels (A-E) or white (F-H). Key: LCW, lateral cell wall; CPl, cell plate; L, lumen; E, epidermis; CPa, cortical parenchyma;

As indicated in earlier studies of root apexes of carrot and onion [48,49], extensin is not present in all primary cell walls, but is targeted to possibly strengthen specific apoplastic regions at different developmental stages [27]. This was further illustrated here using the anti-extensin monoclonal antibody, JIM11, to probe the distribution of extensin in grapevine callus and plantlets (Figure 7).

In agreement with previous results [29], the extensin GvP1 could be detected in the lateral cell walls of grapevine callus by JIM11 (Figure 7A). To test whether the cell plate also contained JIM11-reactive epitopes, thin-slice (0.5 μm) sections of resin-fixed callus were studied, where the cell plate was exposed (Figure 7B). However, no JIM11 epitopes could be detected in this structure. The expression of GvP1 extensin in these cells therefore appears to be restricted to lateral walls.

In grapevine plantlets, JIM11 epitopes were readily detectable in epicotyls, where they were limited to cell corners of cortical parenchyma (Figure 7C). In mature root sections, JIM11 signals were also detected in parenchyma cell-cell junctions, but were restricted to the epidermis and adjacent sub-epidermal cortical layers (Figure 7D). In the root cap, JIM11 epitopes were mostly located in internal cell layers, where they occupied often large intercellular spaces (Figure 7E), but were also clearly present in some cell walls (Figure 7F).

These observations confirm that extensin is targeted in grapevine to specific cell walls and/or cell corners, where it is likely to provide localised, structural support to tissues. The effect of extensin network formation on the hydration level of the extracellular matrix reported here suggests that extensin can provide such support through the dehydration of extracellular materials, with resultant formation of denser and more rigid matrix properties.

## Discussion

We have demonstrated that H_2_O_2 _causes a rapid and marked decrease in the hydration of grapevine callus primary walls, concomitant with a significant decrease in wall thickness. H_2_O_2 _is known to play an important role in regulating extension growth [19,50] and the mechanical properties of tissues [18] by driving (peroxidase-mediated) phenolic cross-linking of wall constituents, but to our knowledge, this is the first report that it can effect rapid changes in primary wall hydration.

An analysis of cell wall proteins of grapevine callus revealed that H_2_O_2_-mediated reduction in wall hydration occurred with a marked increase in extensin network levels from minor levels (< 0.6%) to ca. 6% (w/w) of the cell wall matrix on a dry weight basis. Extensin network formation in these primary walls appears to be formed exclusively from the cross-linking of GvP1 [22,29]. This is supported here by the amplification of a single extensin cDNA from these cells using a heterologous primer corresponding to a common motif in dicotyledon extensins. Two peptide sequences from GvP1 could be mapped to this cDNA, confirming its identity.

GvP1 is an abundant protein which displays properties typical of dicotyledon extensins, including repeats of structural Ser-(Hyp)_4 _motifs, interspersed with short (4-7 aa) Tyr-rich sequences, thought to participate in both intramolecular isodityrosine formation [41,46] and intermolecular extensin oligomerisation [51,52]. MALDI-TOF analyses of GvP1-derived peptides also indicated post-translational modifications typical of extensins, including hydroxylation and arabinosylation of proline residues. These findings initially suggest that extensin network formation could contribute to H_2_O_2_-mediated reductions in the hydration of primary cell walls.

Studies of extensin during seed coat cell maturation [44], of its impact on cellular morphology [6,53] and wall tensile strength [54], have suggested developmental roles for extensin. However, it remained unclear whether the interaction of the network extensin with other matrix polymers exerts any direct and significant rheological effects in the cell wall. Extensin can be secreted at an early stage in wall formation and there is evidence that it provides an essential scaffold for matrix assembly during wall regeneration in tobacco protoplasts [5], or cell plate formation in Arabidopsis [7]. Additionally, the existence of chimeric, extensin-like members of the leucine-rich repeat family of receptor-like kinases, such as LRX1 [55], suggests the means by which extensin network formation could provide molecular cues to regulate the down-stream synthesis and targeting of wall matrix materials. It could be argued, therefore, that the function of network extensin might be limited to providing essential structural, chemical and/or molecular cues for the later and correct incorporation of potentially more rheologically influential, nascent wall materials into the developing matrix.

Here, we have examined the effects of extensin network formation within the primary wall matrix. The experimental system utilised therefore does not provide insight into extensin function during the earliest stages of wall formation. Instead, it can be more easily related to that which occurs in the lateral walls of cells undergoing extension growth or growth cessation, or during the formation of cell-cell adhesions in intercellular corners where, in both cases, extensin is co-/secreted and later integrated as a network within an existing matrix of extracellular polymers.

The use of this approach allowed us to conclude that the formation of the extensin network in the grapevine callus primary cell wall can exert a direct and significant reduction in its hydration. As supported by EM observations of concomitant changes in wall width, the resultant increase in wall density must occur with a significant decrease in polymer separation, with consequences to matrix pore size, polymer mobility and overall wall rigidity.

Understanding the mechanistic basis of rapid, H_2_O_2_-induced reduction in wall hydration would be of considerable interest. The rod-like structure of typical dicotyledon extensins contains periodic, short stretches containing Tyr residues involved in the formation of intra- and inter-extensin cross-links [46,52,56]. These latter are typically separated by 3-6 nm along the extended polypeptide (50-100 nm in length [8,38,57]), and therefore can potentially lead to the formation of a relatively dense protein network. The further oligomerisation of Tyr to the trimer pulcherosine [51] and tetrameric di-isodityrosine [52] might permit a more extensive polymerisation of this network.

The reticulation of wall extensin requires Tyr radical-radical condensation and therefore, a close interaction of extensin polypeptides. Recent work with the amphiphilic *Arabidopsis *extensin, AtEx3, has shown this extensin can form rope-like and dendritic structures at interfaces through the lateral self-association of periodic hydrophilic and hydrophobic motifs [7]. Evidence for lateral associations of tomato extensin was also described previously [38]. Such associations could favour the juxtaposition of Tyr residues from neighbouring extensin monomers and thus facilitate Tyr oligomerisation and the intermolecular cross-linking of an essentially 2D network.

Lateral association of AtEx3-like extensins might occur *in vivo *at lipid-water interfaces, such as at the phragmosome-cytosol interface during the early phase of cell plate formation. However, as shown in Figure 7A-B, GvP1 is not directed to the cell plate, but is secreted into the matrix of lateral walls, where its electrostatic interaction with mobile and charged pectins would likely both dissociate and sterically hinder stable extensin-extensin assemblies. Furthermore, the primary structure of GvP1 appears to lack the high periodicity of sequence repeats required for lateral associations of this type.

Instead, the cross-linking of GvP1 could be facilitated by its loose ionic association and entanglement with the charged pectin network, whose high mobility [31,33] and frequent, transient pore closures could promote extensin-extensin approximations for intermolecular bonding and the formation of an entangled *3D *network within the wall matrix.

Recently, we suggested that the formation of the extensin network could lock pectins into a more tightly packed configuration [29]. This is partially supported by the current data, which indicates that the formation of the GvP1 network can drive a reduction in inter-polymer spacing, with the concomitant extrusion of matrix water into the symplast and/or apoplastic space. Several solid-state NMR studies have also shown that a reduction in wall hydration leaves the thermo-mobility of the relatively rigid cellulose-xyloglucan network largely unaffected, while pectic fractions become less mobile, leading to the production of a more compact wall structure [31-33]. It is therefore likely that the formation of network extensin primarily effects a reduction in pectin mobility and pore size, with consequences to overall matrix hydration, density and rigidity. However, it is clear that the matrix density in extensin network-containing walls is not 'locked', but remains sensitive to pectin charge, although significantly less so relative to control cell walls. This can be seen from their continued ability to demonstrate changes in hydration after the alteration of EIBMP (Figures 5, 6) or counterion levels (KCl; Figure 1).

A possible mechanistic explanation is that the GvP1 network contributes an additional, elastic component to the matrix, thus increasing its Young's modulus and ability to oppose the osmotic pressure generated as a result of electrical disequilibrium between the matrix and external solute (MacDougall et al., 2001b).

Clearly, if the formation of the extensin network can drive decreases in matrix hydration as evidenced here, the network must be formed under strain. This is a conceivable result of forming a 3D network within a hydrated and mobile primary wall matrix, as described above. Such a network could still partially accommodate charge-driven changes in matrix swelling by elastic deformation or relaxation.

Characterising the non-extensin EIBMPs required in this process may also be of interest. However, we suggest that the extracellular proteins involved are likely to be the normal complement of ionically-bound proteins of diverse natures. Many proteins, even those of acidic pI, contain patches of surface contiguous basic residues, which allows their binding to charged pectins [58], thus reducing pectin charge and wall swelling [28,37,59]. Consistent with a non-specific nature for these proteins, we find that the substitution of endogenous EIBMPs with a heat- and DTT-resistant fraction of endogenous grapevine EIBMPs, *Medicago *leaf EIBMPs or poly-arginine were all effective in reducing hydration to control levels, and all could be used to closely mimic H_2_O_2 _effects on wall hydration when added to extensin network-containing walls (Figure 6A).

Recently, we reported that extensin network formation was a major contributory factor in wall resistance to digestion by fungal, lytic enzymes [29]. It seems likely that the effects of extensin network formation on matrix hydration and resistance to lytic enzymes are causally related, since reduced hydration could limit matrix pore size and thus restrict the mobility of lytic enzymes into the wall matrix [60]. However, the effect of extensin network formation on primary cell wall hydration is also likely to play an important role in dicotyledon development.

Extensin is largely expressed in tissues containing primary cell walls [5-7] and a few studies have indicated that this structural protein can be targeted to either lateral walls or cell-cell junctions [27,48,49]. This was confirmed in our study of JIM11 signal distribution in selected tissues of grapevine, where this epitope can be located in the plane of the wall and/or within cell junctions. In all cases, the distribution of extensin strongly suggested its role in providing structural support to tissues, either by reinforcing cell walls or strengthening cell-cell adhesion. Where extensin is present in cell walls, a H_2_O_2_- and extensin network-mediated reduction in matrix hydration could decrease polymer separation with an increase in pectin viscosity. This could provide increased resistance to shear stress between microfibril layers, thus imparting an increased cell wall rigidity and mechanical support to these tissues. Similarly, the formation of the extensin network within intercellular junctions, which contain mainly pectin, could also decrease pectin pore size, with a consequent increase in viscosity to reinforce cell-cell adhesion strength.

Interestingly, our data with primary cell walls indicates that extensin network formation increases matrix density, but also that these walls remain sensitive to changes in matrix charge. This could occur through changes in the interaction of proteins with the extracellular matrix or counter-ion concentration as shown here, but could also occur through developmentally regulated changes in the activities of e.g. plasma membrane proton pumps [61], ion channels [62], or pectin methyl esterases [63]. This suggests that extensin network formation may impart a more rigid structure to the plane of the wall or within intercellular junctions, without compromising the walls ability to undergo controlled swelling to facilitate the incorporation of additional wall material and matrix modifying enzymes required for further wall extension and development.

## Conclusions

We have provided evidence that H_2_O_2 _can drive a rapid reduction in primary wall hydration and wall thickness in grapevine callus cells, and that extensin network formation was the major causal event in this process. These findings confirm an important role for the extensin network in the regulation of primary cell wall density, and demonstrate that extensin effects on wall hydration are sensitive to matrix charge. This report emphasises the importance of considering the effect of endogenous wall proteins on wall properties when extrapolating data from *in vitro *model studies to the interpretation of wall function and properties *in vivo*. The contribution of the extensin network in reducing primary wall hydration may be the principal means by which this structural protein effects changes in the biophysical properties of extracellular materials, with consequences to polymer separation, viscosity, wall rigidity, cellular adhesion and the regulation of extension growth.

## Methods

### In vitro culture of Vitis vinifera cv. Touriga callus and plantlets

Grapevine (*V. vinifera *cv Touriga) callus were maintained on modified MS [64] medium at 25°C in the dark, as described in [22] and subcultured every 3 weeks. Grapevine (*V. vinifera *cv Touriga) plantlets used in immunolocalisation studies were propagated *in vitro *as described previously [65]. Apical leaves were obtained from *Medicago truncatula *cv. Jemalong grown for 4 weeks, as described in [66].

### Isolation of cell walls from grapevine callus cultures

Cell walls from 3 week-old cultures of grapevine callus were prepared as either native cell walls (containing endogenous, ionically binding matrix proteins), or were saline-extracted or H_2_O_2_-treated, essentially as described previously [22]. Briefly, grapevine callus was frozen in liquid nitrogen and milled using a Spex 6700 freezer/mill (Spex Industries, Inc., Edison, NJ) at 360 strokes min^-1 ^for 4 min. Homogenates were dispersed in 5 × vol/g (FWT) of 15 mM sodium acetate (pH 4.5; hereafter suspension buffer) and centrifuged at 4500 *g *for 5 min. The crude wall pellet was then washed in suspension buffer containing 0.1% Triton X-100, followed by two washes in suspension buffer alone by centrifugation. Where saline extraction of EIBMPs was desired, walls were incubated in 2 × volume g^-1 ^(fresh weight) of 1 M KCl at 4°C for 5 min with gentle agitation, followed by two washes in excess suspension buffer by centrifugation. For H_2_O_2 _treatments, isolated cell walls were incubated in 100 μM H_2_O_2 _in suspension buffer for 15 min at 24°C.

### Assays of cell wall hydration

Assays of the cell wall swelling properties as a function of K^+ ^concentration utilised the molecular exclusion of 260,000 Da dextran labelled with fluorescein isothiocynate (FITC; Sigma), as described by [35], except that 2 mL of KCl solution in suspension buffer containing 0.2% w/w FITC-dextran was added to 5 mg (fresh weight) cell walls, and allowed to equilibrate at 20°C for 12-16 hours. Final KCl concentrations of samples were estimated by conductivity (Conductometer, Consort, Lisbon). Each sample was centrifuged and the concentration of FITC-dextran in the supernatant determined from its absorbance at 450 nm. The calculated dilution of the added FITC-dextran correlates with the volume and the mass of cell wall accessible to the probe. The hydrated mass of the cell wall was determined from the difference between the total mass of suspension and the mass of the solution accessible to the probe. Each isolated swelling point represents the average from at least four independent samples, and each was measured in triplicate. Comparative data were analysed by unpaired Student's *t*-test.

### Quantifying GvP1 and EIBMPs abundance in cell walls

To quantify the level of soluble, monomeric extensin in cell walls, saline eluates (1 M KCl in 15 mM sodium acetate (pH 4.5)) of control or H_2_O_2_-incubated grapevine callus or cell walls (derived from 35 mg fresh weight cells) were injected onto a Superose-12 column (HR 10/30, Amersham-Pharmacia Biotech, Uppsala) and quantified as described previously [29]. To estimate the endogenous levels (μg protein.mg^-1 ^cell wall (DW)) of non-GvP1 EIBMPs in saline extracts, the total protein content in saline extracts from cell walls incubated for 2 h in 100 μM H_2_O_2 _was measured with the BioRad protein assay kit (BioRad, Germany). This provided a good estimate of the abundance of non-GvP1 EIBMPs in control cell walls, since such extracts contained only residual monomeric GvP1, and Superose-12 chromatography demonstrated that the sum of A_280 _absorbing materials contributed by non-GvP1 EIBMPs remained unaltered after incubation with H_2_O_2_.

### Altering the content of extracellular, ionically-bound matrix proteins in cell walls

Saline-extracted cell walls were prepared from isolated cell walls of callus as described above. The cell walls were equilibrated in suspension buffer, then incubated at 4° C for ca. 15 minutes with occasional agitation in the presence of selected fractions of EIBMPs (including pure GvP1 and GvEP fractions, described below). Unbound proteins were removed by 2 × washing in excess suspension buffer by centrifugation at 4,500 *g*.

For the preparation of whole saline extracts, three week old cultures of grapevine callus were washed exhaustively with suspension buffer, then gently agitated in 2 × Volume/g (fresh weight) of the same buffer containing 1 M KCl to elute ionically bound cell surface proteins. The eluate was collected by vacuum-assisted ultra-filtration through a 0.45 μM filter (Sartorius) then concentrated and equilibrated in suspension buffer by pressure-assisted filtration through a 10 kDa cut-off membrane (Diaflow, Amicon, Beverly, MA).

Saline eluates of H_2_O_2_-treated callus cells were prepared as described above except that cells were first dispersed in suspension buffer containing 100 μM H_2_O_2 _(2 × vol. g^-1 ^fresh weight) and incubating at 24°C in the dark during 15 minutes with gentle agitation, prior to saline extraction.

To prepare grapevine EIBMPs with depleted levels of GvP1, whole saline eluates were subject to Superose-12 gel filtration chromatography under the conditions described above. Fractions containing GvP1 (eluting at 8.5 - 10.5 mL) were removed and the remaining EIBMPs desalted and concentrated as described above for total saline eluates.

The preparation of *Medicago *leaf EIBMPs was performed as described previously [66], except that saline extracts of isolated leaf walls were equilibrated in 15 mM sodium acetate (pH 4.5) before use.

The reconstitution of grapevine cell walls with endogenous levels of GvP1 and GvEP1 utilised 60 μg of GvP1 and 20 ng of GvEP1 mg^-1 ^(DW) cell wall. Grapevine callus, *Medicago *leaf EIBMPs and poly-L-arginine were added to 20, 20 and 10 μg mg^-1 ^(DW) cell wall, respectively. In all cases, the level of wall-bound EIBMPs and extensin was monitored by Superose-12 chromatography as described above.

### Purification of GvP1

Concentrated and desalted saline eluate of grapevine callus cell walls (described above) was injected onto a 1.5 × 20-cm SP-Sepharose column (Amersham-Pharmacia) equilibrated in 20mM sodium acetate (pH 4.5), and washed with the same buffer at 2 mL min^-1 ^until all non-binding, A_280_- absorbing material had been removed. Bound proteins were then eluted within a 0-0.5 M NaCl gradient over 60 minutes with a flow rate of 2 mL min^-1^. Fractions containing GvP1 were identified by Superose-12 chromatography (elutes at 8.5 mL - 10.5 mL) under conditions described above, then pooled and concentrated by pressure-assisted filtration (10 kDa cut-off; Diaflow, Amicon, Beverly, MA). The concentrate was adjusted to 10% trichloroacetic acid, centrifuged at 12000 *g *and the supernatant diluted in distilled water to ≤ 0.1% TCA, followed by concentration and equilibration in distilled water by pressure-assisted filtration, as above.

### Purification and MALDI-TOF analysis of GvP1 glycopeptides

Pure GvP1 was added to a 70% formic acid solution (0.1:1 w/v) containing cyanide bromide (CNBr) (1:2 w/w) and incubated under a stream of nitrogen at 25°C, for 16 h in the dark. The reaction mixture was then diluted 10 times with pure water, and subject to 2 cycles of freezing, lyophilisation to near-dryness (Speed-Vac, Savant Instruments, Holbrook, NY) and resuspension in pure water to remove volatiles. Peptides were separated by Superose-12 chromatography under conditions described above. A peak eluting at 15-16 mL was applied separately to a 150 × 3.9 mm C_18 _reverse-phase column equilibrated with 0.1% TFA. The column was washed with 5 mL of 0.1% TFA prior to peptide elution within a 0-80% linear acetonitrile gradient in H_2_O over 7.5 mL at a flow rate of 0.5 mL min.^-1^. The eluate was monitored at 280 and 254 nm using a Gold system detector (Beckman, USA). Selected peptide peaks were pooled, desalted by C_18 _Zip-Tips and characterised by MALDI-TOF MS (Bruker model Reflex III) in the linear mode using sinapinic acid as matrix.

### Amino acid compositional analyses, amino acid sequencing

The amino acid composition of pure GvP1 or the saline-insoluble fraction of grapevine cell walls were analysed as described previously [29]. The extensin peptides P4 and P6 were prepared for sequencing by Edman degradation as previously described [22]. The peptides were then sequenced using a Procise™ protein sequencer (Model 491 HT; Applied Biosystem, Warrington, UK) in the gas phase mode from Biobrene™-treated glass fiber discs.

### RNA extraction and cloning of a 5' truncated cDNA of GvP1

Total RNA was isolated from using Qiagen's RNeasy Plant Mini kit, according to manufacturers' instructions. For 3' RACE PCR, first strand cDNA was synthesised from 1 μg total RNA using PoxS3 primer (5'(T)_18 _nn-cac agt agc aac aag tcg gat ccg acc (t)20 (agc) (agct)3') in 25 μl containing 200 U Superscript II (Invitrogen), 0.2 mM dNTPs, 2.5 μl 0.1 M DTT, for 50 min at 42° C. First strand cDNA synthesis was terminated by 15 min heat inactivation at 70°C, then diluted to 50 μl and 2.5 μl used directly as template for PCR amplification using a Mastercycle Personal thermocycler (Eppendorf). PCR utilised the primer PoxS2 (5'-cacagtagcaac aagtcggatccgacc-3') and the primer Ext1 (5'-aa(ag)(at)(gc)icciccicciccigtita(ct)aa(ag)-3'), where i = inosine) corresponding to the common dicotyledon extensin motif KSPPPPVYK. The conditions for PCR cycles were 1 min denaturation at 94°C, 2 min annealing and 2 min extension at 72°C and 30 cycles at 60° C. A final extension of 50 min was utilised to facilitating subsequent TA cloning. The amplification was carried out by 2.5 U Taq Polymerase (MBI Fermentas) in a total volume of 50 μl reaction consisting of 5 pmoles POXS2, 10 pmoles POXS4 0.2 mM of each dNTP, 5 mL 10 × PCR buffer (Stratagene) with (NH_4_)_2_SO_4_.

PCR products were visualized on 1% TAE agarose gel and products 300 bp or larger were purified (Agarose Gel DNA Extraction kit, Roche), ethanol precipitated and ligated into pGEM T Easy vector (Promega). JM109 competent cells were transformed with the ligation products as per manufacturers' instructions. Ten positive clones were selected and sequenced.

### Analysis of cell wall monosaccharide composition

All the cell wall samples for sugar analysis were freeze-dried and dispersed in 72% H_2_SO_4 _for 3 h at room temperature. After dilution to 2N H_2_SO_4 _samples were hydrolysed for 1 h and 2.5 h at 100°C. Uronic acids were assayed by the calorimetric method and neutral sugars by gas-chromatography [35].

### Measurement of cell wall thickness by fast-freeze scanning electron microscopy

Control or H_2_O_2_-treated callus cells were either homogenised or equilibrated for 30 min in 0, 15 or 100 mM KCL solution and immediately frozen by plunging into liquid nitrogen slush at -210°C and then fractured in the scanning electron microscope. Micrographs were taken at 5 kV or below, using a Philips/FEI XL30 field emission SEM (FEI UK Ltd., Cambridge, UK) fitted with a CT1500HF cryo-system (Gatan UK, Oxford, UK). Samples were routinely sputter-coated with 2 to 4 nm of platinum before imaging. Measurements of cell wall thickness were made directly on samples in the microscope using the Philips imaging software. Each preparation was searched for the best aligned sections, which looked precisely perpendicular to the cell wall plane, and the width of no more than 3 readings were taken from the same preparation. The average wall widths were calculated from at least 10 replicates after the identification and elimination of Tukeys outliers. Significance tests utilised unpaired Students t-test.

### Immunolocalisation of JIM11 epitopes

Fresh grapevine callus was frozen in liquid nitrogen then sectioned to 20 μm with a Leica CM3050 S cryostat. Materials from *in vitro *cultured grapevine plantlets were immersed into fixative solution of 4% paraformaldehyde, 0.1% glutaraldehyde in 0.1 M sodium phosphate buffer, pH 7.4, subject to 10 min of vacuum-infiltration and placed at 4°C over night with gentle agitation. The samples were then dehydrated in an increasing ethanol series before embedding in LR White resin at room temperature and polymerization at 60°C for 24 h. The sections were cut to 0.5 μm using a Leica ultra-microtome and collected on poly-L-lysine (Sigma-Aldrich) glass slides. The sections were incubated for 8 h at 4°C with 5% PBS/milk containing a 5-fold dilution of the primary antibody, JIM11 [48]. After washing with PBS, a 100-fold dilution of the secondary antibody (Cy5 conjugated goat anti-rat IgG (Jackson Immunoresearch) was added to the section for 1 h in darkness. The slides were incubated with a 1% Calcoflour white in PBS before washing in PBS and mounting in Slow Fade Light anti-fade kit (Molecular Probes). Sections were observed with a Leica TCS SP5 II confocal microscope and images processed with ImageJ.

## Authors' contributions

CSP performed the majority of the hydration analyses, the preparation of extensin, general cell wall extracts and cell walls, GvP1 peptide purification and contributed to the writing of the manuscript. JMLR assisted in the production of callus and the preparation of general cell wall extracts. ADV was responsible for cloning 5'-truncated cDNA sequences of GvP1 and for immunocytochemical techniques. KF undertook electron microscopy of wall fragments at John Innes Center, UK. AJM hosted CSP at the Institute of Food Research, UK, during the initial part of this work and contributed expertise in cell wall hydration analyses. PAPJ contributed to the production of general grapevine and Medicago materials, hydration analyses, the interpretation of MALDI spectra, the conception, design and coordination of the study. All authors have read and approved the final manuscript, and declare that they have no competing interests.

## Supplementary Material

Additional file 1 **Superose-12 analysis of the binding of EIBMPs to saline-extracted walls**. The chromatographic traces represent extracts from saline-extracted cell walls (35 mg (FW) equivalent) after incubation with: A) endogenous levels of whole grapevine EIBMPs, B) endogenous levels of Pure GvP1 + GVEP1, C) endogenous levels of non-extensin EIBMPs, D) 20 μg EIBMPs from Medicago leaf. In all cases, traces depict bound EIBMPs before (upper trace) and after (lower trace) 30 min incubation with H_2_O_2_. The arrows (A, B) depict a reduced content of monomeric GvP1. Click here for file

Additional file 2 **The effect of EIBMPs and poly-L-argine on the hydration of saline-extracted walls**. All measurements were made and expressed as described in Figure 5. Values for saline-extracted cell walls (□) and native cell walls (■) are shown for reference. Note that the addition of ca. 20 μg Medicago and grapevine EIBMPs or ca. 10 μg poly-L-arginine to these walls reduces hydration to native cell wall levels (control). Click here for file
