# Supplementary figures and images for: Extensin network formation in Vitis vinifera callus cells is an essential and causal event in rapid and H2O2-induced reduction in primary cell wall hydration
Source: BMC Plant Biol. 2011 Jun 14;11:106. doi: 10.1186/1471-2229-11-106 (PMC3141637; doi:10.1186/1471-2229-11-106)

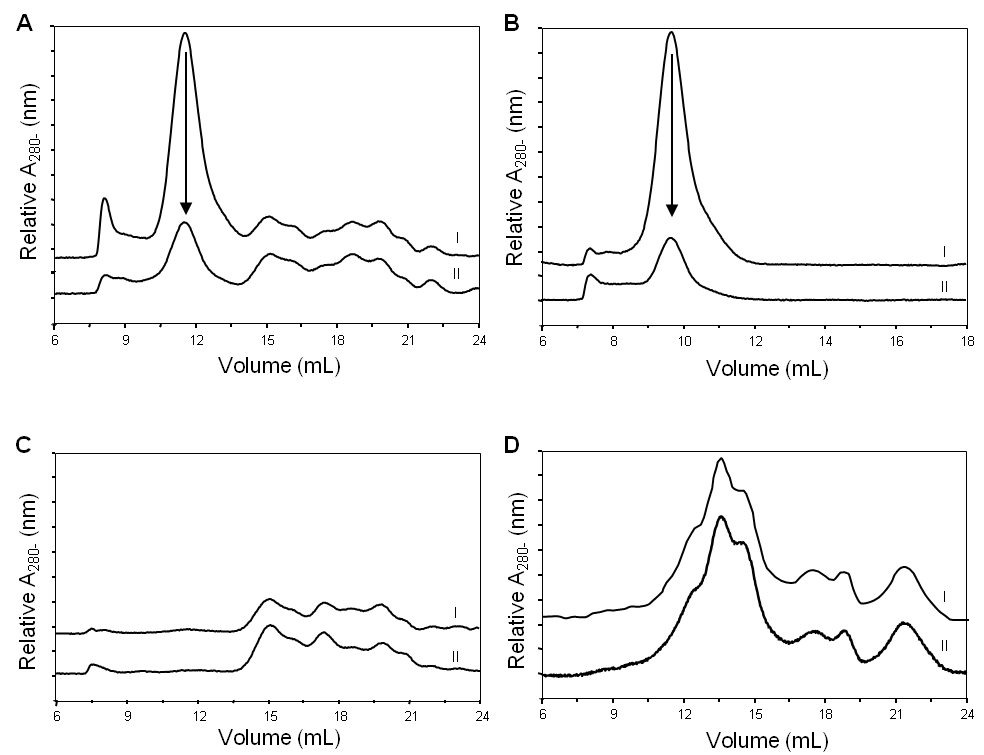

Supplement: Additional file 1 — Superose-12 analysis of the binding of EIBMPs to saline-extracted walls. The chromatographic traces represent extracts from saline-extracted cell walls (35 mg (FW) equivalent) after incubation with: A) endogenous levels of whole grapevine EIBMPs, B) endogenous levels of Pure GvP1 + GVEP1, C) endogenous levels of non-extensin EIBMPs, D) 20 μg EIBMPs from Medicago leaf. In all cases, traces depict bound EIBMPs before (upper trace) and after (lower trace) 30 min incubation with H2O2. The arrows (A, B) depict a reduced content of monomeric GvP1. [file 1471-2229-11-106-S1.PNG]

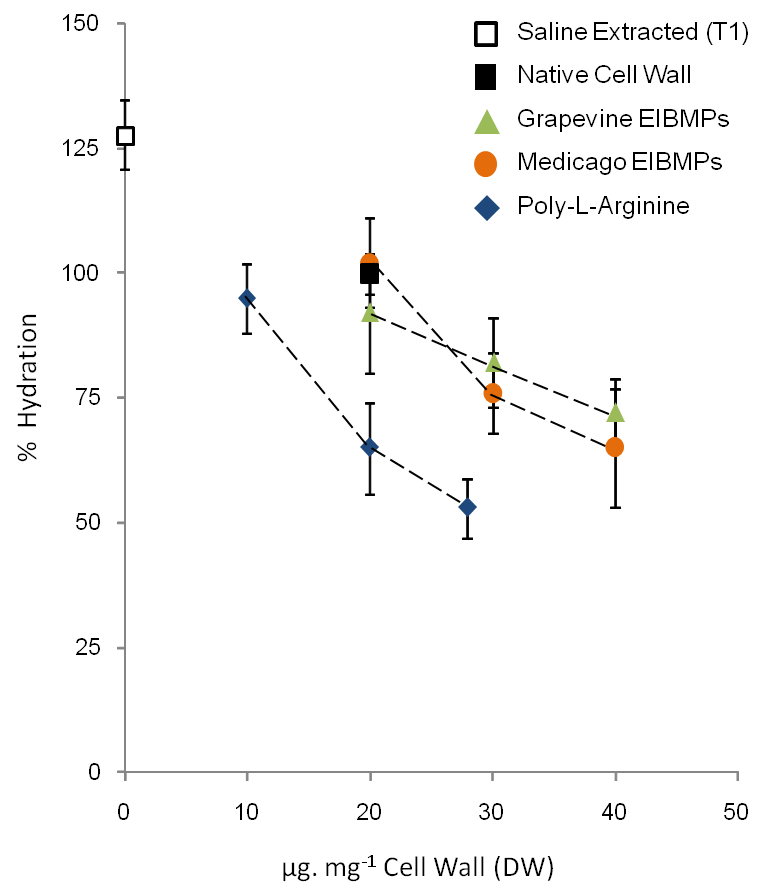

Supplement: Additional file 2 — The effect of EIBMPs and poly-L-argine on the hydration of saline-extracted walls. All measurements were made and expressed as described in Figure 5. Values for saline-extracted cell walls (□) and native cell walls (■) are shown for reference. Note that the addition of ca. 20 μg Medicago and grapevine EIBMPs or ca. 10 μg poly-L-arginine to these walls reduces hydration to native cell wall levels (control). [file 1471-2229-11-106-S2.PNG]
